# Gastrostomy Tube Insertion Complications and Patient Care Outcomes in a Tertiary Care Hospital

**DOI:** 10.7759/cureus.18458

**Published:** 2021-10-03

**Authors:** Faisal Alhasani, Salem Bazarah, Mohammad Ahmed, Basim Alraddadi, Amjad Alotaibi

**Affiliations:** 1 Gastroenterology, King Abdulaziz University Faculty of Medicine, Jeddah, SAU

**Keywords:** complications, endoscopy, outcome, retrospective study, percutaneous endoscopic gastrostomy

## Abstract

Background

Percutaneous endoscopic gastrostomy (PEG) is a widely known procedure where an endoscopist inserts a tube through the stomach to provide enteral nutrition. The existing literature shows inconsistent results regarding complication rates, and very few studies have examined the relationship between patient characteristics and PEG outcomes. Therefore, we aimed to investigate PEG tube insertion outcomes and determine different variables associated with these outcomes.

Methods

This retrospective record review included 207 patients who underwent PEG tube insertion at King Abdulaziz University Hospital (KAUH), Jeddah, Saudi Arabia, between 2010 and 2021. We obtained variables such as demographics, complications, and length of hospitalization. The Student t-test, chi-square test, and Mann-Whitney test were used in the data analysis.

Results

Of 207 patients, 106 were male (51.2%). The patient's median age was 10 years, and the median length of hospital stay was two days. The PEG-related complication rate was 32.4%, while the 1-year adverse outcome rate was 44.9%. The most common complications were unspecified fever (21.3%) and vomiting (14%). We found a significant relationship between dysphagia and length of hospitalization (P=0.015) and between age and the occurrence of tube leakage (P=0.021). Another significant relationship was found between the number of PEG insertions and gastrostomy-site infection (P=0.046).

Conclusions

This study's results indicate the importance of a thorough review of patients' medical records; some patient characteristics can be valuable predictors of PEG outcomes. Thus, we urge physicians to study each patient to anticipate PEG tube insertion outcomes carefully. Moreover, we recommend that researchers with access to larger patient registries study more variables to reach unified guidelines that ensure the best possible outcomes.

## Introduction

Over the decades, the medical field has been improving procedures and techniques, one of which is gastrostomy tube insertion. In 1980, Gauderer published his newly developed technique, percutaneous endoscopic gastrostomy (PEG); the aforementioned endoscopic approach was primarily designed as an alternative to the previously preferred surgical approach [1]. After that, it became prevalent, and currently, it is one of the most prevalent procedures used to satisfy the nutritional requirements of patients who cannot ingest food orally [2]. During this procedure, an endoscopist uses upper endoscopy to insert a tube with two openings, one outside over the skin and the other opening facing the stomach, to secure access for long-term tubal feeding in specific patient populations that require enteral access [3]. Many patients who cannot maintain sufficient oral nutritional intake may require PEG, including patients with neurological dysfunctions, such as cerebral palsy, stroke, neuromuscular disorders, and patients who have had head and neck trauma and upper aerodigestive tract surgeries will benefit from such procedure [4].

Although PEG is generally considered a safe procedure, there are many complications associated with it; these complications, such as bleeding, wound infection, ileus, and necrotizing fasciitis, can occur during or even after the procedure in the short- or long term [4]. Complications can be classified according to severity, either major or minor; major complications include aspiration pneumonia and haemorrhage, whereas minor complications include PEG-site infection and minor bleeding [5]. One study of 1041 patients reported a 30-day mortality rate of 5.8% [6], while another study published in 2012 showed complication rates as high as 39% at two weeks after the procedure and 27% at two months after the procedure [7]. The inconsistency in the previously mentioned complication rates in the literature and the insufficient number of studies conducted here in the middle east and Saudi Arabia call for more studies and investigations tackling this topic. Accordingly, our primary objective was to investigate the complications and outcomes associated with the PEG procedure. Our secondary objective was to determine what comorbidities and demographic-if any-can affect the procedure outcome at our tertiary care centre.

## Materials and methods

Study design and population

After obtaining ethical approval from the ethical committee of the College of Medicine of King Abdulaziz University Hospital (KAUH), Jeddah, Saudi Arabia (reference number: 110-21), we started conducting the study from March 2021 to June 2021. We retrospective reviewed and included all patients who underwent PEG tube insertion with a valid date of PEG tube insertion during 2010-2021. Therefore, 207 patients were included to investigate the complications and outcomes associated with the PEG procedure performed in KAUH, a tertiary care centre.

Data collection

Furthermore, we wanted to determine what comorbidities and demographics-if any-can affect the outcome of the procedure at our tertiary care centre. Thus, we started manually obtaining the following data from our hospital information system (Phoenix): patient medical record number, date of birth, sex, weight, height, date of admission, date of discharge, date of procedure, comorbidities, complications following the procedure, number of PEG procedures, indications for PEG, abnormal findings during the procedure, chief complaint, and vitals.

Statistical analysis

Continuous variables are presented as means±standard deviation for descriptive statistics, and they were compared using the Student t-test. Categorical variables are summarized using number and frequency, and the chi-square test was used to compare them. For non-parametric data, the Mann-Whitney test was used to perform bivariate analysis. Data were entered into Google Forms and then further extracted into Excel, version 16.0 (Microsoft Corp., Redmond, WA) and finally analyzed utilizing SPSS for Windows, version 21.0. (IBM Corp., Armonk, NY). A P-value <0.05 was considered significant.

## Results

Overall, 229 patients underwent the PEG procedure at our hospital between 2010 and 2021. Twenty-two patients missing the procedure date were excluded from the study; accordingly, 207 patients were included. The study patients' demographics and characteristics are summarized in table 1. Of 207, 106 were male (51.2%), and 101 were female. There were 118 (57%) patients younger than 18 years of age and 89 patients aged 18 years or older. Patients' median age was 10 years (range: 0-94 years). Among the patients, 104 were outpatients, and 103 were inpatients. Cerebral palsy (33.8%) was the most common indication to perform PEG in our centre, followed by neurological diseases (17.9%) and cerebrovascular diseases (14%). The most common chief complaint that led to PEG tube insertion was dysphagia (28%), followed by poor oral intake (26.6%) and poor weight gain (19.8%). Hypertension (24.2%), diabetes mellitus (19.3%), and cerebral palsy (11.1%) were the most common comorbid conditions among our patients. Additionally, the median length of hospital stay after performing the procedure was two days (range: 0-237 days). Regarding the 1-year adverse outcomes (summarized in table 2), fever (21.3%) was the most common, followed by vomiting (14%), shortness of breath (10%), and aspiration pneumonia (9.7%). Moreover, we found that 93 patients developed at least one 1-year adverse outcome (complication rate=44.9%). We also calculated a PEG-related complication rate of 32.4%, which included bleeding at the procedure site, perforation, PEG-site infection, fever, tube leakage, tube dislodgement, tube obstruction, and aspiration pneumonia.

**Table 1:** Demographics and characteristics of the 207 patients. PEG- Percutaneous Endoscopic Gastrostomy

Demographics and characteristics of the 207 patients.	
Sex, n (%)	
Male	106 (51.2)
Female	101 (48.8)
Median Age (years)	10 (range: 0­94)
Age Group, n (%)	
≥18 years	89 (43)
<18 years	118 (57)
Median Length of Hospital Stay After Performing the PEG Procedure (days)	2 (range: 0­237)
Average Height, cm	118.8±37.9
Average Weight, kg	34.1±30.3
Patient Type, n (%)	
Outpatient	104 (50.2)
Inpatient	103 (49.8)
Indication, n (%)	
Cerebral palsy	70 (33.8)
Neurological diseases	37 (17.9)
Cerebrovascular disease	29 (14)
Head and neck cancer	10 (4.8)
Chief Complaint, n (%)	
Dysphagia	58 (28)
Poor oral intake	55 (26.6)
Poor weight gain	41 (19.8)
Esophageal reflux	10 (4.8)
Vomiting	9 (4.3)
Comorbid Disease, n (%)	
Hypertension	50 (24.2)
Diabetes mellitus	40 (19.3)
Cerebral palsy	23 (11.1)
Seizures	21 (10.1)
Dyslipidemia	15 (7.2)

**Table 2:** Frequency and percent of 1-year adverse outcomes following percutaneous endoscopic gastrostomy tube insertion. PEG- Percutaneous Endoscopic Gastrostomy; MI- Myocardial Infarction

Frequency and percent of 1-year adverse outcomes following percutaneous endoscopic gastrostomy tube insertion.		
1-year Adverse Outcome	n	%
Fever	44	21.3
Vomiting	29	14
Abdominal pain	23	11.1
Shortness of breath	21	10.1
Aspiration pneumonia	20	9.7
PEG tube-site infection	19	9.2
PEG tube leakage	15	7.2
Diarrhoea	12	5.8
Cough	11	5.3
Sepsis	9	4.3
Constipation	9	4.3
PEG tube displacement	7	3.4
Nausea	7	3.4
Bleeding	6	2.9
Aspiration	5	2.4
Altered mental status	4	1.9
Convulsions	4	1.9
Hypoxemia	2	1
Hypoventilation	2	1
Perforation	1	0.5
Arrhythmia	1	0.5
MI	1	0.5
PEG tube obstruction	1	0.5
Acute pancreatitis	1	0.5
Hematemesis	1	0.5
Bronchiolitis	1	0.5

Our analysis found that patients with cerebral palsy had a higher incidence of fever as an adverse outcome after PEG insertion (P<0.000). We also found that the length of hospital stay after PEG tube placement increased if the patient complained of poor oral intake (P=0.015). However, the length of hospitalization decreased if the patient complained of dysphagia (P=0.031) or had hypertension as a comorbidity (P=0.011). Furthermore, we found a significant relationship between the number of PEG procedures performed and the development of fever (P=0.006) and PEG tube-site infection (P=0.046) as adverse events. Moreover, we found significant relationships between age and PEG tube leakage (P=0.021) and between age and altered mental status as an adverse outcome (P=0.015). In addition, the younger the age, the lengthier duration of hospitalization after PEG insertion was expected (P=0.000) (R=-0.481). On the other hand, we found no relationship between age and the incidence of PEG tube dislodgment (P=0.539) or international normalized ratio level and incidence of PEG site bleeding (P=0.263).

## Discussion

In our study, we found that the 1-year adverse outcome rate was 44.9%. This surprisingly high percentage could be credited to the fact that these adverse outcomes may not directly relate to the PEG insertion. Therefore, we calculated a more specific complication rate using only the complications we could relate to the procedure: bleeding at the site of the procedure, perforation, PEG-site infection, fever, tube leakage, tube dislodgement, tube obstruction, and aspiration pneumonia. The specific PEG-related complication rate was 32.4% which is comparable to that reported in the literature. Figueiredo et al. reported a minor complication rate of 31% [8]. In addition, Mansoor et al.'s study reported a complication rate of 25% among patients with cancer [9]. Other studies conducted in the United States and Sweden found complication rates of 47% (at 30 days) and 39% (at 14 days), respectively [7,10]. In contrast, Tokunaga et al. and Lee et al. reported complication rates as low as 16.7% and 13.2%, respectively [11,12]. We believe that this variation in the rate of complications could be because of the difference in patient characteristics, e.g., age, ethnicity, comorbidities, and availability and affordability of healthcare, as here in our centre and other governmental centres in Saudi Arabia, a more significant complication rate is expected because of free healthcare. Therefore, patients would not hesitate to return to the hospital regardless of the type of complication.

In our investigation, the most common 1-year adverse event was fever with an unspecified source (21.3%). Similarly, a study from Korea found that fever was the most common complication (3.5%) [11]. Moreover, previous studies conducted in Australia and Germany found that the most common complication among patients was PEG-site infection (10.6% and 10.2%, respectively), which is comparable to our result (9.2%) [13, 14]. Conversely, a study conducted in the United Arab Emirates found aspiration pneumonia to be the most common complication among patients (128 of 362 admissions) [5]. Additionally, a Columbian study found gastrointestinal symptoms (diarrhoea and distention, 32.9%) to be the most common complication [15]. The difference in the results could be attributed to the fact that we assessed the 1-year adverse outcomes in our study. In contrast, the other studies mentioned above-evaluated complications that occurred within a substantially shorter time.

The median length of hospitalization in our study was two days. Similarly, a study conducted by Fortunato et al. reported a median hospital stay of 3 days [16]. However, studies conducted in the United States and Germany reported median lengths of hospital stay of 13.5 and 11 days, respectively [13,17]. The difference between the results could be because of the much higher median and mean ages in the studies mentioned above than in our study (74 and 56 years, respectively, versus 10 years in our study). Moreover, 50% of our patients were outpatients and had a short 1-day admission, which contributed to a shorter length of hospitalization, unlike the American study, which comprised only inpatients.

Cerebral palsy (33.8%) was the most common indication for PEG tube insertion among our study patients. Other studies conducted by Gumaste et al. and Atencio et al. found stroke to be the most common indication (30% and 32.89%, respectively) [15,17]. However, in a study conducted in Australia, head and neck cancer (35.3%) was the most common indication for PEG tube insertion [14]. We believe that the difference between the results could be due to the high percentage of patients younger than 18 years of age included in our study compared to those mentioned above.

Our study found that dysphagia as a chief complaint was associated with a shorter length of hospitalization (P=0.031). In contrast, Fortunato et al. found that patients with preoperative dysphagia had a longer median length of stay of 8 days compared with three days in patients without dysphagia (P<0.00001) [16]. We believe this difference may be because of different interpretations of the word dysphagia, especially for different age groups. Pediatric patients are less likely to verbally complain of dysphagia as it sometimes can only be assumed by the parents. We think these significant relationships between chief complaints and outcomes can be valuable predictors of PEG insertion outcomes. They can be found by using easy-to-access data such as patient notes.

Furthermore, in our study, we found a significant relationship between age and the occurrence of PEG tube leakage. This association could be because the patient's immune system is weaker in the extremes of age, which increases the likelihood of developing local infection and further delaying the wound healing, contributing to PEG tube leakage. Similarly, a Korean study reported that older age and diabetes are risk factors associated with chronic complications of PEG tube insertion, including PEG tube leakage and wound infection [18].

Limitations

The present study has several limitations: The main limitation of this study was its retrospective nature and the lack of essential variables available in the hospital database system, such as the duration of the procedure, type of prophylactic antibiotic used, and type of PEG technique applied; There was a lack of comparative data from Saudi Arabia and studies that investigated the relationship between comorbidities and PEG outcomes worldwide; As we currently do not have any patient registries that combine the data of different hospitals, we had a smaller sample size than that of other studies that used such patient registries.

## Conclusions

We found some interesting significant relationships between age and length of hospital stay, between age and PEG tube leakage, and between the length of hospital stay and hypertension. These results indicate the importance of thoroughly reviewing the patients' medical records because age, comorbidities, and chief complaints could be valuable predictors of patient care outcomes. Therefore, we urge physicians to carefully assess each patient to understand the PEG tube insertion outcomes better beforehand. Moreover, we recommend that researchers with access to larger patient registries conduct studies that include more variables, such as the technique applied to insert the tube, types of antibiotics administered before and after the procedure, and resulting nutritional gain from the procedure, so that we can reach unified guidelines that ensure the lowest possible complication rate and the best outcomes for patients.
